# A comparison of suit dresses and summer clothes in the terms of thermal comfort

**DOI:** 10.1186/2052-336X-11-32

**Published:** 2013-12-19

**Authors:** Can Ekici, Ibrahim Atilgan

**Affiliations:** 1 Department of Mechanical Engineering, Gazi University, Ankara, Turkey

**Keywords:** Thermal comfort, PMV, PPD, Clothing

## Abstract

**Background:**

Fanger’s PMV equation is the result of the combined quantitative effects of the air temperature, mean radiant temperature, relative air velocity, humidity, activity level and clothing insulation.

**Methods:**

This paper contains a comparison of suit dresses and summer clothes in terms of thermal comfort, Fanger’s PMV equation. Studies were processed in the winter for an office, which locates in Ankara, Turkey. The office was partitioned to fifty square cells. Humidity, relative air velocity, air temperature and mean radiant temperature were measured on the centre points of these cells. Thermal comfort analyses were processed for suit dressing (I_cl_ = 1 clo) and summer clothing (I_cl_ = 0.5 clo).

**Results:**

Discomfort/comfort in an environment for different clothing types can be seen in this study. The relationship between indoor thermal comfort distribution and clothing type was discussed. Graphics about thermal comfort were sketched according to cells.

**Conclusions:**

Conclusions about the thermal comfort of occupants were given by PMV graphics.

## Background

### Introduction

Thermal comfort can be defined as the satisfaction of the mind in a thermal environment [1]. Physical and mental productivity of human are increased in this satisfied environment.

The main purposes of the HVAC systems are acceptable comfort and acceptable indoor air quality for human occupants [2]. Engineers have been studying to develop into more comfortable environments for many years. Heating systems and air conditioning systems are utilized to reaching for optimum thermal comfort conditions. If the energy consumption of heating and air conditioning will be decreased, the energy sources can be saved more.

Thermal comfort is a function of air temperature, mean radiant temperature, air velocity, humidity, activity level and clothing thermal resistance. The combined quantitative effects of all parameters were not known until P.O. Fanger’s PMV equation [1]. Predicted Mean Vote (PMV) is a parameter that indicates how the occupants judge the indoor climate. The percentage of people dissatisfied (PPD) can be found by PMV [3]. PMV shows the degree of the environment’s comfort. Thermal comfort distribution can help to giving information about the infiltration points of the rooms.

In this study, thermal comfort analyses of an office in Ankara were processed in winter conditions for summer clothes (Icl = 0.5 clo) and suit dresses (Icl = 1.0 clo). Discomfort or comfort status of the office can be seen on the results for different clothing types. The relationship between indoor thermal comfort homogeneity and clothing type was discussed.

### Studies in literature

Fanger has developed a mathematical model which is named PMV (Predicted Mean Vote). This model predicts the thermal comfort as a function of activity, clothing, air velocity, humidity, mean radiant temperature and air temperature [4].

Fanger has studied on human requirements in future air-conditioned environments. Better air quality is an important factor for higher productivity. Small amounts of clean air should be served where it is consumed, close to the breathing zone of each person [2].

Toftum, Fanger and Jorgensen have studied on the upper limits of air humidity for preventing warm respiratory discomfort. Five different values of skin moisture were analysed in this study. In all experiments, the combination of humidity, environmental parameters and clothing parameters were controlled. Relative humidity of the skin is an important parameter for an occupant who is exposed to sunlight directly. A mathematical model was given in their studies [5].

Olesen has studied on the international standards and the ergonomics of the thermal environments. The standards include evaluation methods for moderate, hot, and cold environments [6,7].

Fanger and Toftum have studied on the extension of the PMV model to non-air-conditioned buildings in warm climates. For warm climates, occupants may feel different than the PMV predicts in non-air-conditioned buildings. Fanger and Toftum suggest an extended PMV model that includes an expectancy factor for non-conditioned buildings in warm climates [8].

Gadi has developed a new computer program, which was coding for the prediction of human thermal comfort. It incorporates six thermal comfort indices. The indices are “Fanger’s Comfort Equation”, “Sharma’s Tropical Summer Index” and “Madsen’s Equivalent Temperature” [9].

Yao, Li and Liu have developed a new theoretical PMV model that is called aPMV (Adaptive Predicted Mean Vote). The aPMV model can be described as aPMV = PMV/(1 + λ × PMV). The equation gives the generic relationship between the Adaptive Predicted Mean Vote (aPMV) and the Predicted Mean Vote (PMV) in free-running buildings [10].

Rowe has studied on the office occupants’ thermal comfort for a building in Sydney. In this study, thermal comfort analysis was processed for different gender groups, different activity rates, and different temperatures [11].

Ampofo, Maidment and Missenden have studied on the thermal comfort for underground railway environments of London. In this study, old railway tunnels and modern railway tunnels were compared in the terms of thermal comfort. Both of the tunnels’ air velocity values were acceptable. The air temperature was high especially at the old deep line tube station. The relative humidity across the network was not measured. Air humidity values were assumed %50 in PMV and PPD calculations. In general the predicted values of thermal sensation matched quite well with the perceptions of the people interviewed [12].

## Methods

### Theory

**Figure 1:**
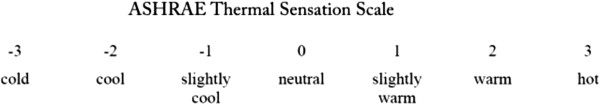
ASHRAE Thermal sensation scale.

PMV (Predicted Mean Vote) equation was developed by P.O.Fanger in 1970s [7,13-15]. Comfort criteria were described by theoretical, experimental and statistic studies of P.O.Fanger [14,15]. PMV equation provides a score that corresponds to the ASHRAE Thermal Sensation Scale. ASHRAE Thermal Sensation Scale is shown in Figure 1[16]. In this scale, zero is the best condition for the PMV values, the minus values connote to cool and cold environments and the positive values of PMV indicates to warm and hot environments. PMV equation gives a score about thermal comfort. When the PMV score converges to zero, the thermal environment is comfortable for maximum occupants (i.e. if PMV = 0, about %95 of the all occupants are pleased in this thermal environment) [16,17].

**Figure 2:**
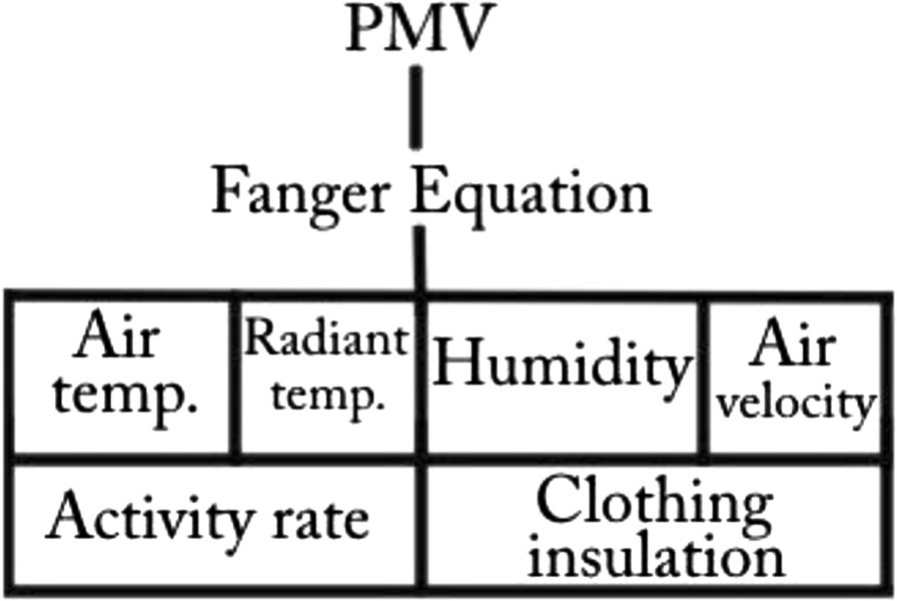
PMV equation’s variables.

PMV equation’s variables are shown in Figure 2. These variables are mean radiant temperature, air temperature, relative humidity, air velocity, activity rate and clothing insulation [18-20].

PMV equation is shown in equation 1.

(1) $$\begin{array}{ll} \mathrm{PMV}= & \left(0.352*e^{-0.042\left(\frac{M}{A_{\mathrm{Du}}}\right)}+0.032\right)\\ & *\left[\frac{M}{A_{\mathrm{Du}}}*\left(1-\eta \right)-0.35*\left[43-0.061*\right)\right)\left(\frac{M}{A_{\mathrm{Du}}}*\left(1-\eta \right)-p_{a}\right]\\ & -0.42*\left[\frac{M}{A_{\mathrm{Du}}}*\left(1-\eta \right)-50\right]-0.023*\frac{M}{A_{\mathrm{Du}}}\left(44-p_{a}\right)-0.0014\\ & *\frac{M}{A_{\mathrm{Du}}}*\left(34-T_{a}\right)-3.4*10^{-8}*f_{\mathrm{cl}}\\ & *\left[{\left(T_{\mathrm{cl}}+273\right)}^{4}-{\left(T_{\mathrm{mrt}}-273\right)}^{4}\right]-\left(f_{\mathrm{cl}}*h_{c}*\left(T_{\mathrm{cl}}-T_{a}\right)\right] \end{array}$$

where

(2) $$\begin{array}{ll} \;T_{\mathrm{cl}}= & 35.7-0.032*\frac{M}{A_{\mathrm{Du}}}*\left(1-\eta \right)-0.18*I_{\mathrm{cl}}\\ & *\left[3.4*10^{-8}\right)*f_{\mathrm{cl}}*{\left[\left(T_{\mathrm{cl}}+273\right)\right)}^{4}-\left({\left(T_{\mathrm{mrt}}+273\right)}^{4}+f_{\mathrm{cl}}*h_{c}\left(T_{\mathrm{cl}}-T_{a}\right)\right] \end{array}$$

where

(3) $$h_{c}=\left\{\begin{array}{l} 2.05*{\left(T_{\mathrm{cl}}-T_{a}\right)}^{0.25}>10.4\sqrt{v}\to h_{c}=2.05{\left(T_{\mathrm{cl}}-T_{a}\right)}^{0.25}\\ 2.05*{\left(T_{\mathrm{cl}}-T_{a}\right)}^{0.25}<10.4\sqrt{v}\to h_{c}=10.4\sqrt{v} \end{array}\right\}$$

Predicted Percentage Dissatisfied (PPD) was developed by P.O.Fanger. This index predicts the percentage of unsatisfied people who are likely to be dissatisfied with a thermal environment [18]. PPD equation is shown in equation 4.

(4) $$\mathrm{PPD}=100-95*e^{-\left(0.03353*\mathrm{PM}V^{4}+0.2179*\mathrm{PM}V^{2}\right)}$$

**Figure 3:**
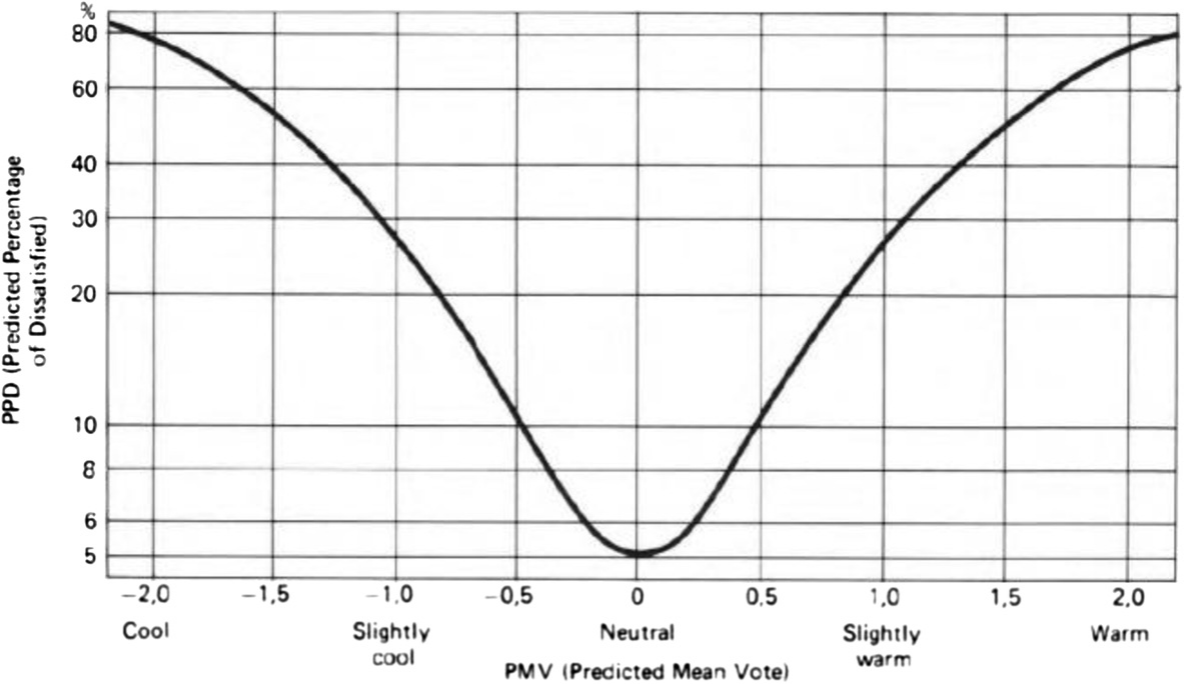
**The relationship between PPD and PMV**[1]**.**

The approximate relationship between PPD and PMV is shown in Figure 3. When PMV value converges to zero, PPD value decreases. The relationship can be seen by the parabolic line in Figure 3.

The PMV-PPD limits are suggested by ASHRAE in a standard for evaluating moderate thermal environments. It is recommended to use

(5) -0.5<PMV<+0.5

(6) PPD<%10

limits for an acceptable thermal environment.

### Experiment

**Figure 4:**
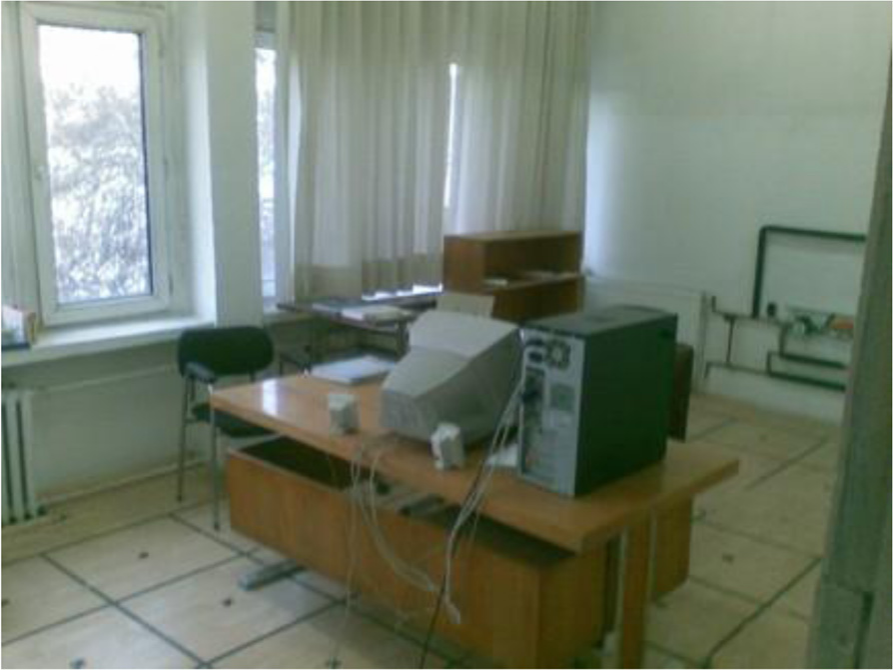
The experimental room.

The experimental room is heated from radiator and it is located in Ankara, Turkey. The room’s area is 23.4 m^2^; also the room has got two windows which are located on the east. Dimensions of the each window are (277 * 142) cm^2^. One of the windows was closed with a curtain. The room and the room’s windows, curtains, the details of the room can be seen in Figure 4.

**Figure 5:**
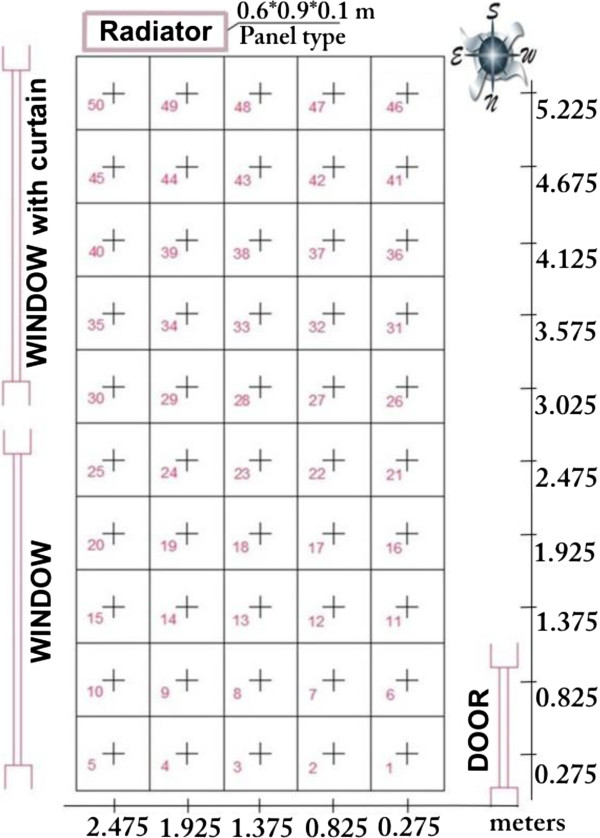
The experimental room’s cells.

The room was divided to fifty square cells as shown as in Figure 5. The variables were measured from the centre points of these cells. PMV values were calculated for each cell’s centre points for 0.2 meter, 0.6 meter and 1 meter heights separately. Square cells are 55*55 cm^2^.

**Figure 6:**
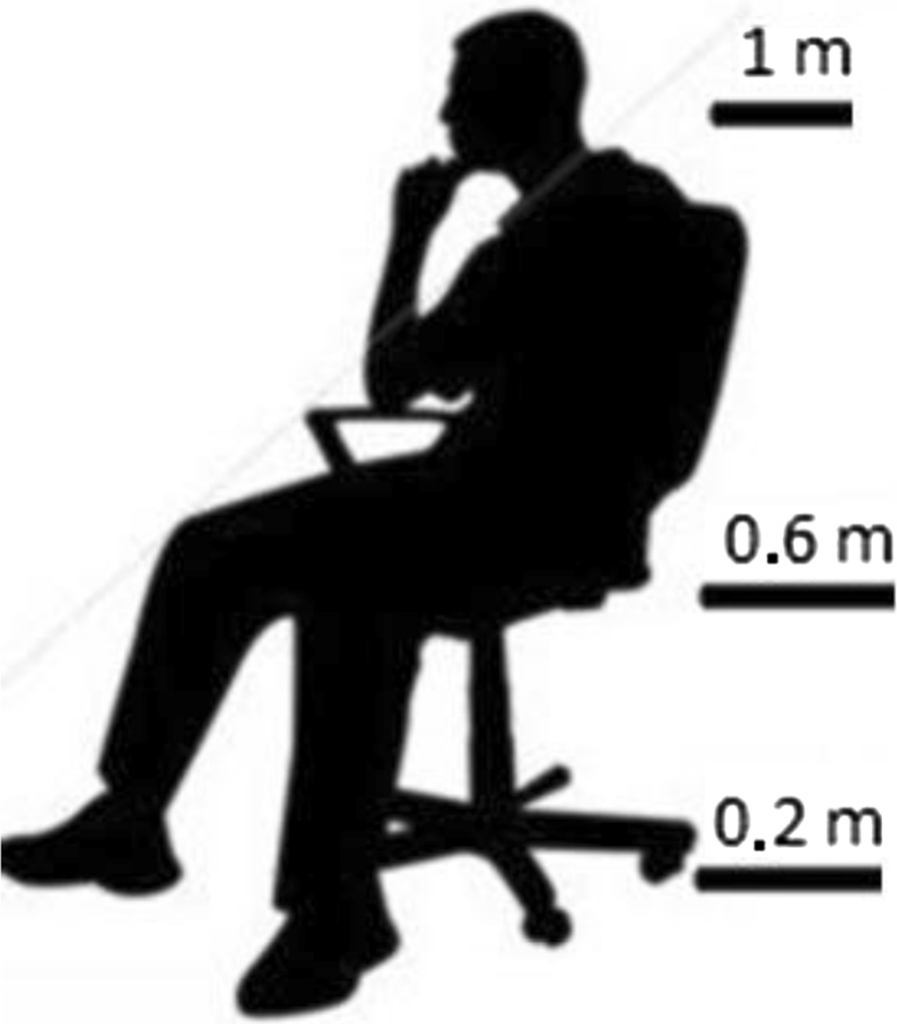
Heights of measurement for an office occupant.

It is suitable to measure variables for 0.2, 0.6 and 1 meter heights from the floor to determine PMV values for an office occupant who sits on an office desk [4]. A sitting occupant and the measurement points for this occupant can be seen in Figure 6. In experiment; air temperature, mean radiant temperature, relative air humidity and air speed were measured from 0.2, 0.6 and 1 meter heights for each cell along two hours. PMV values were calculated for these heights on computer software. PMV values can be seen on graphics for the different points of room.

Clothing area factor (f_cl_), thermal insulation of clothing (I_cl_), surface area of human body (A_DU_), metabolic rate production (M), and mechanic efficiency (η) can be seen on Table 1.

**Table 1:** Variables in calculations

		**M/A**_**DU **_**(kcal/hm**^**2**^**)**	**f**_**cl**_	**I**_**cl **_**(clo)**	**η**
Office occupant	Suit dress	60	1.15	1	0
	Summer cloth	60	1.1	0.5	0

**Figure 7:**
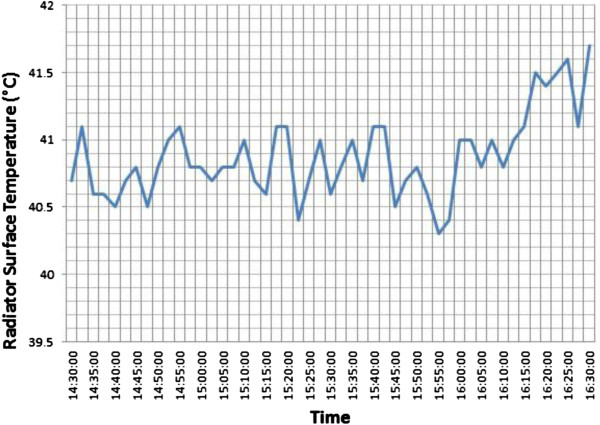
Radiator surface temperatures (°C).

The heating system of the office has got a boiler. An electric resistance heats the water up to 60°C in the boiler. Hot water comes from the boiler to the radiator via pipes. Surface temperatures of the radiator along the experiment are shown in Figure 7 (22 January 2011, between 02.30 pm. - 04.30 pm). Surface temperatures of the radiator surface were between 40°C and 42°C. There were not big fluctuations between the surface temperatures of the radiator during the experiment. The differences between the radiator’s surface temperatures were negligible and may not affect to the thermal comfort. The highest difference between the surface temperatures of the radiator is 1,4°C in this study. The surface temperatures were measured by an infrared thermometer. In primary calibration laboratories, the best measurement uncertainty of the infrared thermometer can be 0,8 ~ 1,0°C.

Ethic note: Any human or animal subjects were not used in the experiments.

The weather conditions in Ankara at 22 January 2011 are shown in Table 2.

**Table 2:** Weather conditions in Ankara at 22 January 2011*

**Mean temperature**	**Maximum temperature**	**Minimum temperature**
3°C	9°C	- 3 (°C)

*http://www.wunderground.com (IBEYTEPE2 weather station).

### Procedures and application

**Figure 8:**
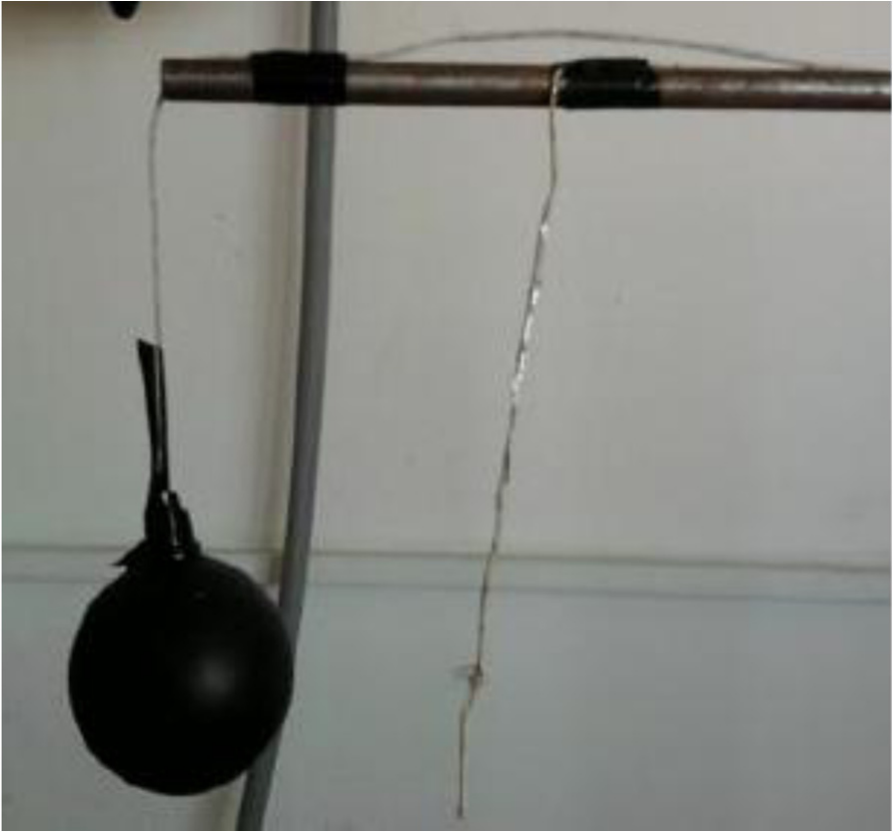
Black globe and thermocouples.

**Figure 9:**
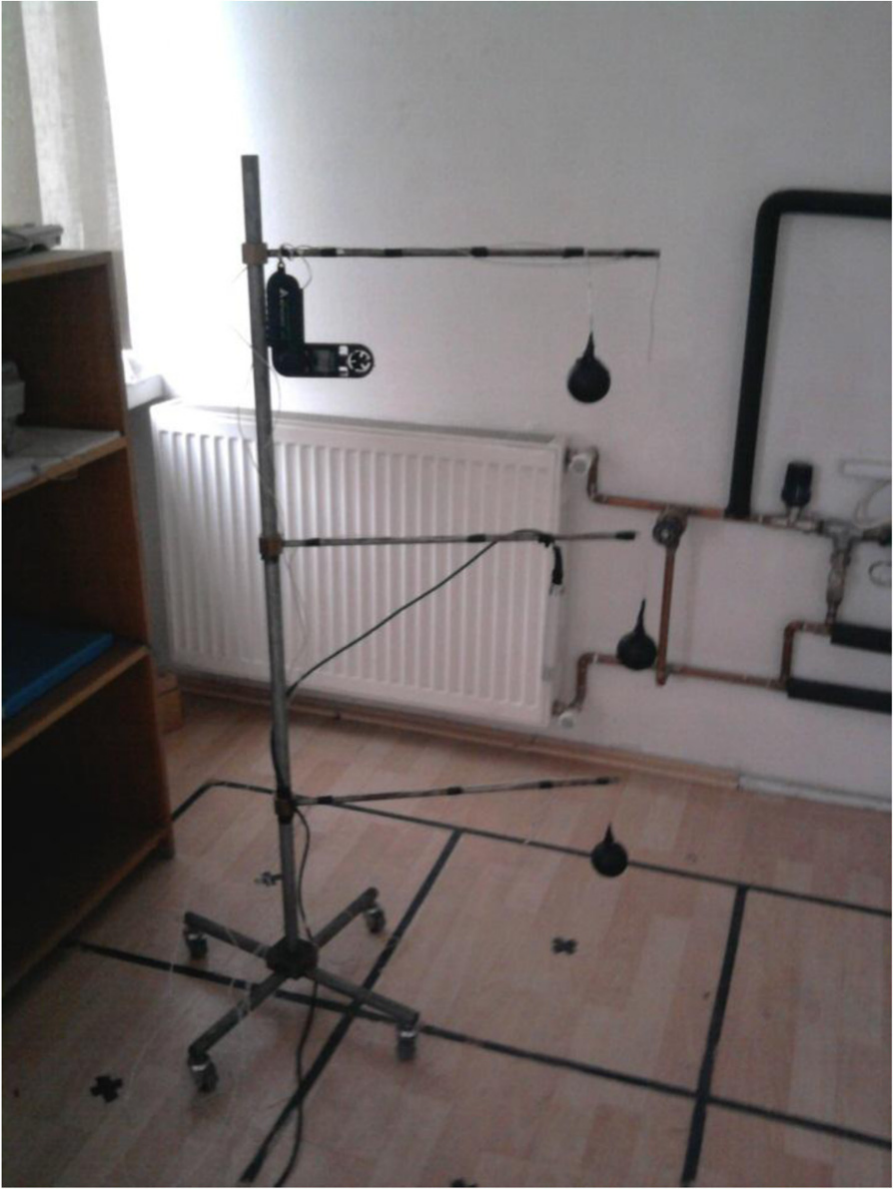
Measuring setup.

Air temperature (T_a_), mean radiant temperature (T_mrt_), relative air velocity (ʋ) and relative air humidity (w) are measured. The measurement devices are computer controlled humidity probe, temperature data logger, anemometer. Air temperature was measured by thermocouples which are connected to the data logger. Relative air velocity was measured by anemometer. Mean radiant temperature was measured by a black globe thermometer. Thermocouple was installed in the black globe. All of the devices were calibrated. The accuracies of the devices are ± 2 %RH, ± 0,3°C, 0,1 m/s. The black globe and thermocouple can be seen on Figure 8. The measuring setup is shown in Figure 9.

Metabolic rate production (M) and mechanic efficiency (η) values were selected from the tables. Metabolic rate production values and mechanic efficiency for the experiment is shown in Table 1.

Saturated vapour pressure (P_g_) was taken from the thermodynamic charts. Water vapour pressure (P_a_) can be calculated by relative air humidity and saturated vapour pressure.

Thermal resistance of clothing (I_cl_), clothing area factor (f_cl_) is given on Table 1.

Surface temperature of clothing (T_cl_) was calculated by iterative methods with computer based software. The values in the previous steps were processed in PMV equation. PMV values were calculated by computer software. This software is based on Visual Basic. Iterations were made by the computer software. This computer software was developed by Can Ekici.

## Results

**Figure 10:**
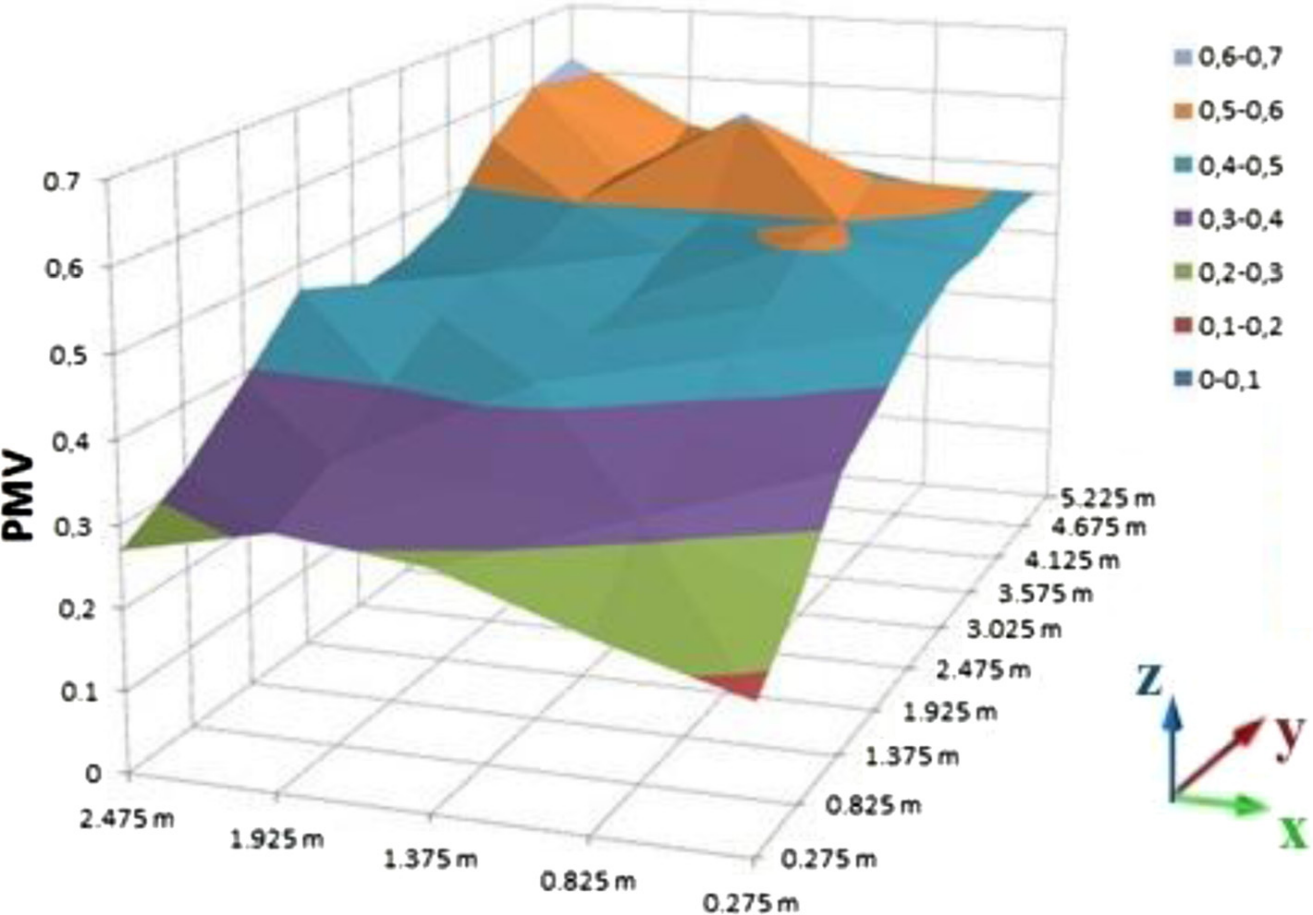
PMV values for suit dress, 0.2 meter heights.

**Figure 11:**
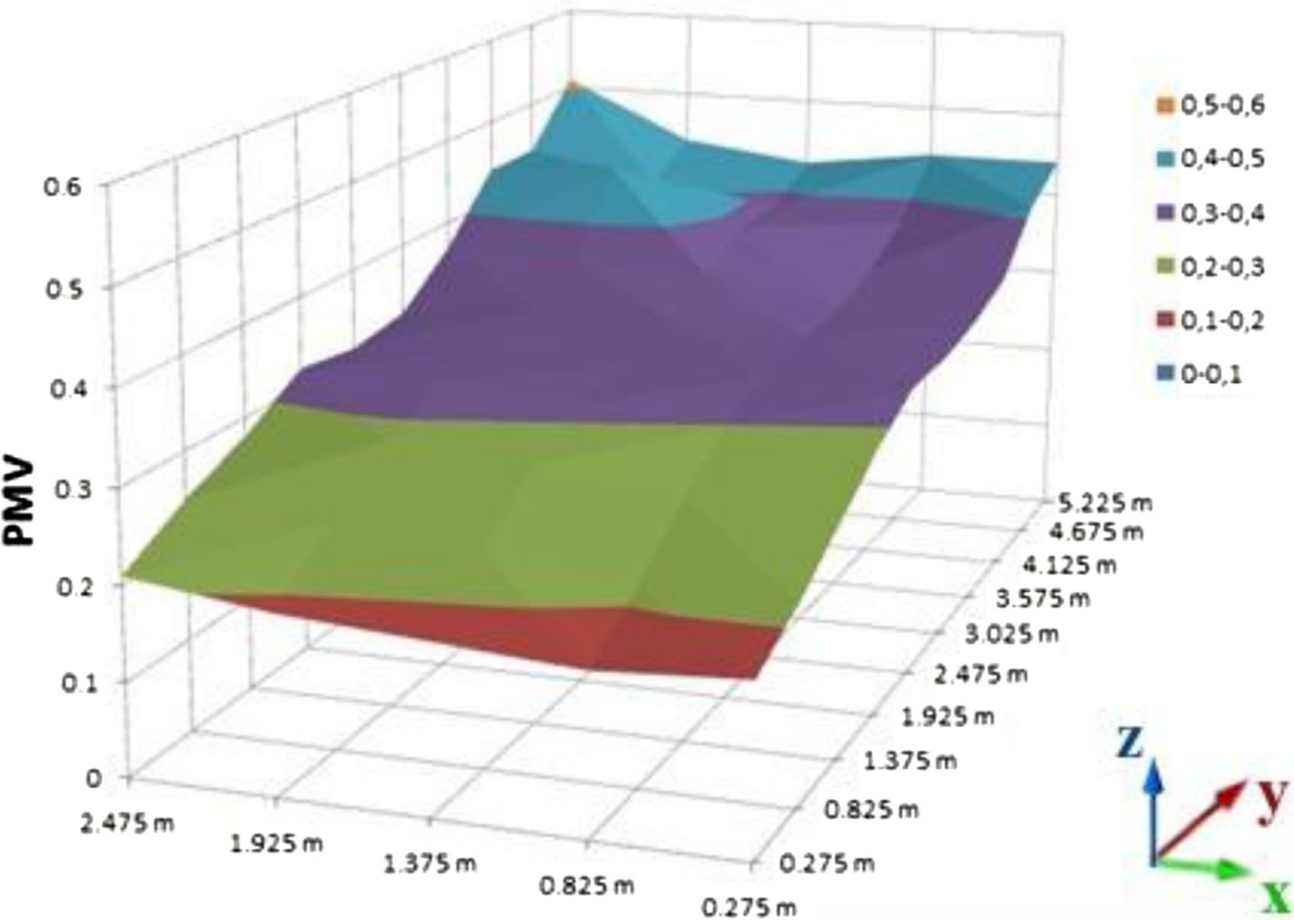
PMV values for suit dress, 0.6 meter heights.

**Figure 12:**
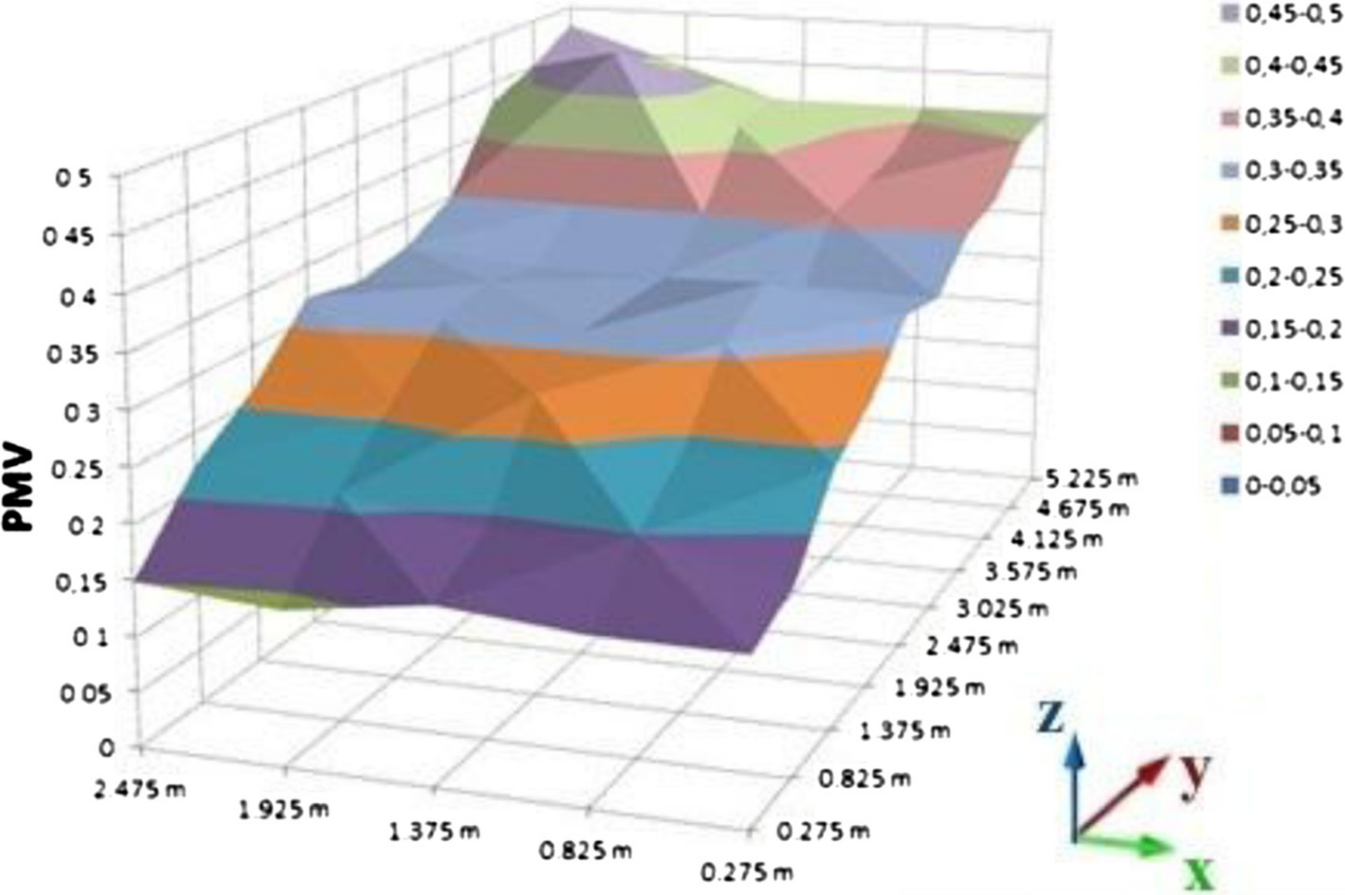
PMV values for suit dress, 1 meter heights.

PMV calculations of room were processed on the computer platform. As the result of these calculations, graphics were sketched on MS Office Excel as a distribution of the room. Different graphics are given for 0.2, 0.6 and 1 meter heights (Figures 10, 11, and 12 for suit dresses). PMV results for the suit dresses for 0,2 meter can be seen on Figure 10. PMV values of 0,6 meter for suit dresses are shown in Figures 11 and 12 shows PMV values of 1 meter height. In Figure 10, the PMV values are greater than the PMV values on the Figures 11 and 12. Because, 0,2 meter points are closer to the radiator than 0,6 and 1 meter points. The points in the 0,6 meter are closer to the radiator than the points in the 1 meter. Cause of that, in some cells the PMV values for 0,6 meter is greater than the PMV values in 1 meter height. All of the PMV values are positive for suit dresses in these three figures. An occupant who wears suit may feel slightly warm in this environment. Mean PMV and PPD values of the cells in the room for suit dresses are shown on Table 3.

**Table 3:** PMV and PPD for suit dress

**Heights from floor (meters)**	**Mean PMV**	**Mean PPD (%)**
0.2	0.436	8.965
0.6	0.322	7.156
1	0.313	7.036

**Figure 13:**
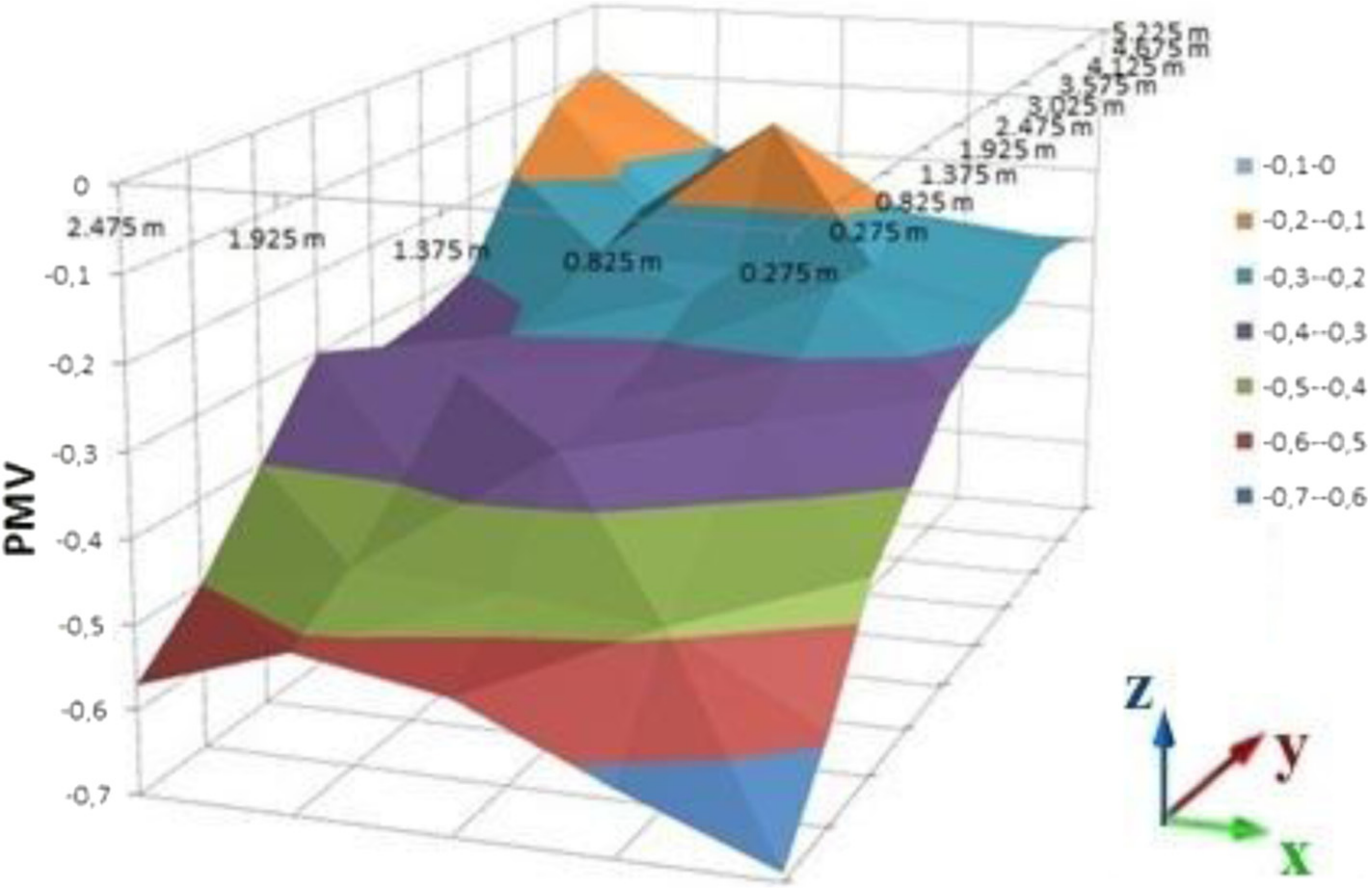
PMV values for summer cloth, 0.2 meter heights.

**Figure 14:**
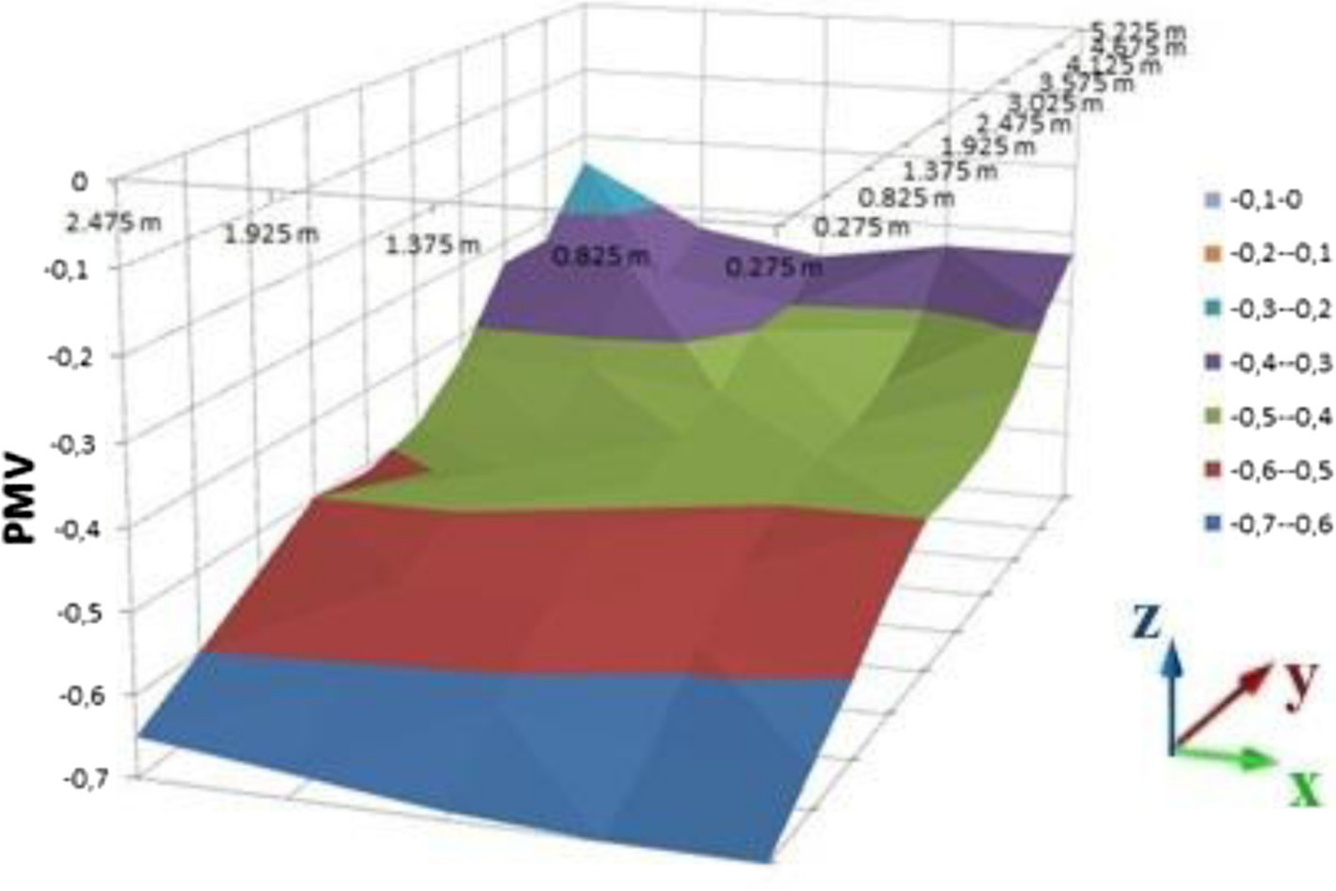
PMV values for summer cloth, 0.6 meter heights.

**Figure 15:**
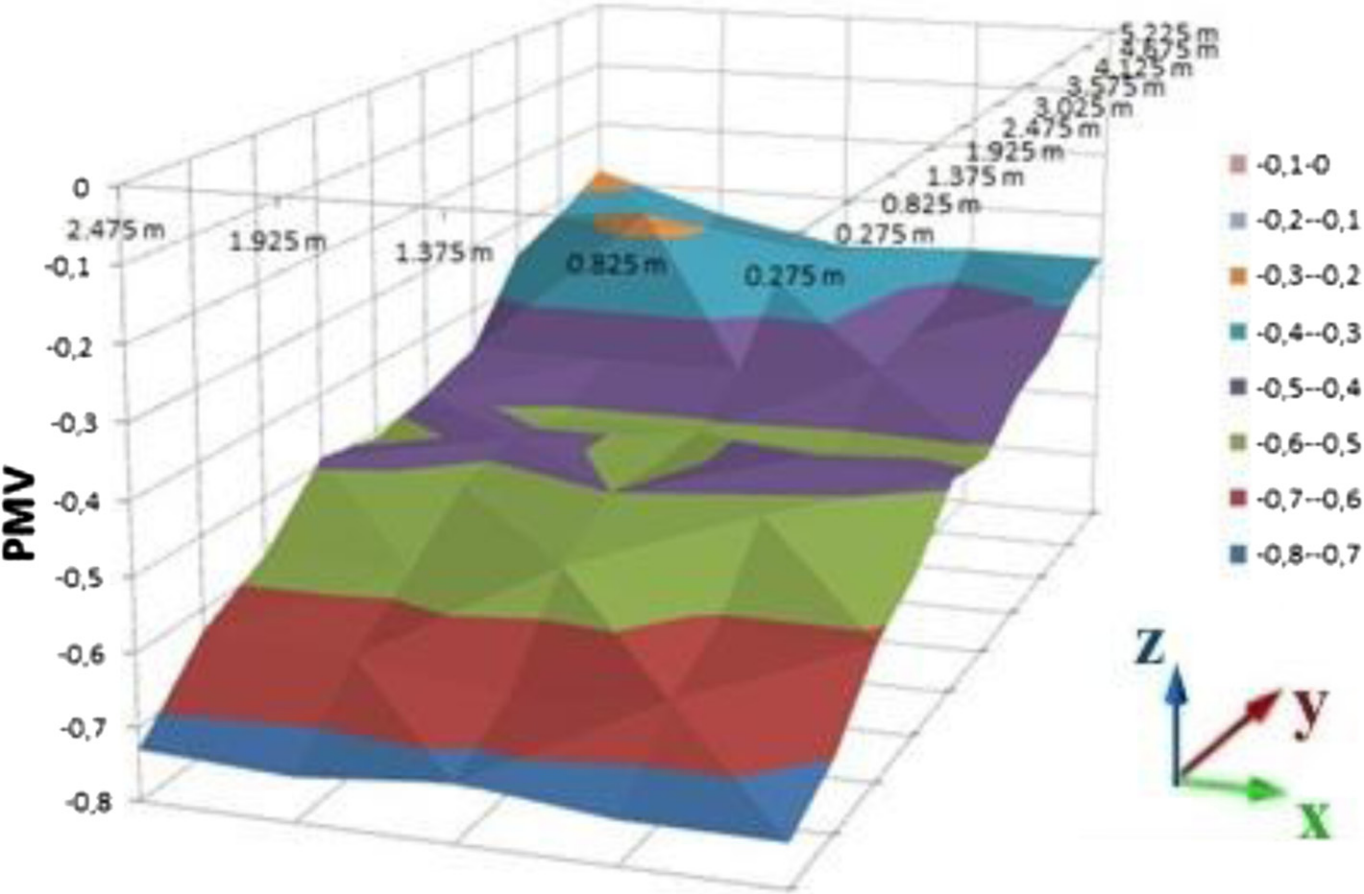
PMV values for summer cloth, 1 meter heights.

Mean PMV and PPD values of the cells in the room for summer clothes are shown on Table 4. PMV and PPD graphics are shown on Figures 13, 14 and 15 as distribution of the room for 0.2, 0.6 and 1 meter heights. Figure 13 is the graphic of the 0,2 meter height, Figure 14 is for 0,6 meter and Figure 15 is for 1 meter heights. For summer clothes, the PMV values in Figure 13 are higher than the PMV values in the other figures as suit’s graphics. All of the PMV values for summer clothes are negative in the three figures. An occupant who wears summer clothes in this environment may feel slightly cool.

**Table 4:** PMV and PPD for summer clothes

**Heights from floor (meters)**	**Mean PMV**	**Mean PPD (%)**
0.2	- 0.337	7.362
0.6	- 0.494	10.099
1	- 0.506	10.352

## Discussion

Mean values of PMV and PPD is acceptable for ASHRAE limits. The mean PMV values for suit dress are between neutral and slightly warm. PMV values in the closer cells to the radiator are greater than the PMV values in the other cells. It may be caused by infiltration and insufficient insulation. PMV values in the closer cells to the door are lesser than the PMV values in the other cells. For the suit dress, almost all of the PMV values are acceptable.

Mean values of PMV and PPD are near to acceptable limits for summer clothes. The mean PMV values for summer cloth are between neutral and slightly cool.

Distribution of the PMV values in the room is slightly nonhomogeneous, it caused by the radiator’s location and infiltration. It is similar in the graphics of suit dress. The cells which are far away from the radiator cannot be heated as well as the cells near to the radiator.

## Conclusions

1. If it is enough to wearing clothes that have greater I_cl_ values instead of setting thermostat degree to higher temperatures, the occupants can feel the environment more comfortable without saving energy. Cost analysis can provide information about energy saving.

2. Distribution of the PMV values in the room is slightly nonhomogeneous. It is due to the location of the radiator and infiltration. Infiltration can be caused by insufficient insulation of windows and the door. This situation is not related to type of clothing. This problem can be solved by using systems that heat the environment more homogenously (i.e. floor heating system). A study about it can be found in literature [21].

3. Both of the distributions of PMV values in room for suit dresses and summer clothes are close to the acceptable limits of ASHRAE Standards. A thermal environment can be comfortable for an occupant who wears suit dresses (I_cl_ = 1.0 clo), and for another occupant who wears summer clothes (I_cl_ = 0.5 clo). Thermal comfort in an environment can be provided for different wearing types.

4. Correct selection of the cloth is one of the most important factors for the comfort. The selection of the cloth is important for the thermal comfort. Energy consumption can be minimized.

5. This study can be developed for new type of clothes. For example; new generation working cloths’ thermal comfort analysis can be processed by this method.

6. For winter conditions, summer clothes may increase the level of human discomfort in the non-insulated environments that are not heated homogenously. Summer clothes can be more acceptable for the environments which are heated homogenously. In this study, comfort level of the occupant who wears summer clothes, is more acceptable in the cells that are near to the radiator. Suit dress may be more preferable than the summer cloth for an environment that is not heated homogenously in winter conditions.

## Abbreviations

PMV: Predicted Mean Vote; M: Metabolic rate production, units of kcal/h; ADU: Surface area of human body, units of m^2^; η: Mechanic efficiency; Pa: Water vapour pressure, units of mmHg; Ta: Air temperature, units of°C; fcl: clothing area factor; the ratio of the surface area of the clothed body to the surface area of the naked body; Tcl: Surface temperature of clothing, units of°C; Tmrt: The mean radiant temperature, units of°C; hc: Convective heat transfer coefficient, units of (kcal/m^2^h°C); Icl: Thermal resistance of clothing, units of clo (1 clo = 0.155 m^2^K/W); ʋ: Relative air velocity, units of m/s; PPD: Predicted Percentage Dissatisfied, units of %.

## Competing interests

The authors declare that they have no competing interests.

## Authors’ contributions

CE and IA were modeling the study and environment. CE was developing a computer software for the calculations. The data was processed and graphics were sketched by CE. IA participated in the reviewing of the manuscript. Both authors read and approved the final manuscript.
